# Hearing Loss, Tinnitus, and Dizziness in COVID-19: A Systematic Review and Meta-Analysis

**DOI:** 10.1017/cjn.2021.63

**Published:** 2021-04-12

**Authors:** Zahra Jafari, Bryan E. Kolb, Majid H. Mohajerani

**Affiliations:** Department of Neuroscience, Canadian Centre for Behavioural Neuroscience, University of Lethbridge, Lethbridge, Canada

**Keywords:** Hearing loss, Tinnitus, Dizziness, Vertigo, Otologic symptoms, Coronavirus, SARS-CoV-2

## Abstract

**Objectives:**

Extensive studies indicate that severe acute respiratory syndrome coronavirus (SARS-CoV-2) involves human sensory systems. A lack of discussion, however, exists given the auditory–vestibular system involvement in CoV disease 2019 (COVID-19). The present systematic review and meta-analysis were performed to determine the event rate (ER) of hearing loss, tinnitus, and dizziness caused by SARS-CoV-2.

**Methods:**

Databases (PubMed, ScienceDirect, Wiley) and World Health Organization updates were searched using combined keywords: ‘COVID-19,’ ‘SARS-CoV-2,’ ‘pandemic,’ ‘auditory dysfunction,’ ‘hearing loss,’ ‘tinnitus,’ ‘vestibular dysfunction,’ ‘dizziness,’ ‘vertigo,’ and ‘otologic symptoms.’

**Results:**

Twelve papers met the eligibility criteria and were included in the study. These papers were single group prospective, cross-sectional, or retrospective studies on otolaryngologic, neurologic, or general clinical symptoms of COVID-19 and had used subjective assessments for data collection (case histories/medical records). The results of the meta-analysis demonstrate that the ER of hearing loss (3.1%, CIs: 0.01–0.09), tinnitus (4.5%, CIs: 0.012–0.153), and dizziness (12.2%, CIs: 0.070–0.204) is statistically significant in patients with COVID-19 (*Z* ≤ −4.469, *p* ≤ 0.001).

**Conclusions:**

COVID-19 can cause hearing loss, tinnitus, and dizziness. These findings, however, should be interpreted with caution given insufficient evidence and heterogeneity among studies. Well-designed studies and follow-up assessments on otologic symptoms of SARS-CoV-2 using standard objective tests are recommended.

## RÉSUMÉ

Pertes auditives, acouphènes et étourdissements chez des patients atteints de la COVID-19 : une étude systématique et une méta-analyse.

**Objectifs:**

Des études approfondies indiquent désormais que le syndrome respiratoire aigu sévère lié au coronavirus (SRAS-CoV-2) peut affecter les systèmes sensoriels humains. L’impact du virus sur les systèmes auditif et vestibulaire est tel qu’on peut considérer qu’il existe à l’heure actuelle un manque de discussions quant à cet impact. L’étude systématique et la méta-analyse qui suivent ont été effectuées afin de déterminer justement la fréquence des pertes auditives, des acouphènes et des étourdissements causés à partir de 2019 par le SRAS-CoV-2.

**Méthodes:**

Nous avons interrogé certaines bases de données (PubMed, ScienceDirect, Wiley) mais aussi les mises à jour de l’Organisation mondiale de la santé (OMS) au moyen des mots-clés combinés suivants : « COVID-19 », « SRAS-CoV-2 », « pandémie », « dysfonction auditive », « pertes auditives », « acouphènes », « dysfonction vestibulaire », « étourdissements », « vertiges » et « symptômes otologiques ».

**Résultats:**

Au total, 12 articles ont satisfait à nos critères d’admissibilité et ont été inclus dans cette étude. Ces articles étaient prospectifs et basés sur un seul groupe, de nature transversale, de nature oto-rhino-laryngologique et rétrospective, de nature neurologique ou axés sur les symptômes cliniques généraux de la COVID-19. Tous ont recouru à des évaluations subjectives dans la collecte des données (historique des cas, dossiers médicaux). Les résultats de la méta-analyse ont ainsi démontré que les fréquences de pertes auditives (3,1 %, IC : 0,01-0,09), d’acouphènes (4,5 %, IC : 0,012-0,153) et d’étourdissements (12,2 %, IC : 0,070-0,204) étaient statistiquement significatives chez des patients atteints de la COVID-19 (Z ≤ −4,469, *p* ≤ 0,001).

**Conclusions:**

La COVID-19 peut entraîner des pertes auditives, des acouphènes et des étourdissements. Ces résultats devraient toutefois être interprétés prudemment étant donné le manque de preuves et l’hétérogénéité des articles en cause. Des études bien conçues et des évaluations des symptômes otologiques du SRAS-CoV-2 lors de suivis à l’aide de tests standardisés objectifs sont donc recommandées.

## Introduction

During the past two decades, several viral epidemics such as the severe acute respiratory syndrome coronavirus (SARS-CoV), H1N1 influenza, and the Middle East respiratory syndrome coronavirus (MERS-CoV) have been reported.^1,2^ On January 30, 2020, the World Health Organization (WHO) officially declared the epidemic caused by SARS-CoV-2, CoV disease 2019 (COVID-19), as a public health emergency of international concern.^1,2^ SARS-CoV-2, which is a novel CoV from the same family, presents flu-like symptoms. The polymerase chain reaction (PCR) is a diagnostic test of COVID-19, which consists of the collection of upper respiratory samples via nasopharyngeal/oropharyngeal swabs.^2^ The disease contains nonspecific symptoms, and its presentation ranges from no symptoms to severe pneumonia and death. Typical signs and symptoms based on confirmed PCR, which generally develop 5–6 d after infection (range 1–14 d), consist of fever, dry cough, fatigue, sputum production, shortness of breath, sore throat, headache, dizziness, myalgia or arthralgia, chills, nausea or vomiting, nasal congestion, diarrhea, hemoptysis, and conjunctival congestion.^3,4^

SARS-CoV-2 infection can lead to a wide range of extrapulmonary, sensory, and neural complications, such as sudden onset olfactory and/or gustatory dysfunction,^5,6^ otologic symptoms,^7–9^ nonspecific symptoms, and long-term neurological complications.^10^ It has been shown that the neuroinvasion driven by SARS-CoV-2 is associated with the angiotensin-converting enzyme 2 (ACE2) mechanism, as a functional receptor for the virus.^11^ This enzyme receptor is commonly found in lung type 2 alveoli. It is also expressed by many cells, including glial cells and neurons, and can cause neurological involvement through direct or indirect mechanisms.^12,13^ Given the suspicion of COVID-19 is mostly based on its typical symptoms, patients with early-onset sensory-neural manifestations such as hearing loss, tinnitus, and/or dizziness/vertigo may be misdiagnosed, which can cause the further spread of the virus. Likewise, the timeline of virus development from initial symptoms to moderate or severe complications, roughly five days, is long enough for the virus to enter and damage the brainstem cranial nerves and nuclei.^12^ Despite extensive research and several meta-analyses on olfactory, gustatory,^5,6,14^ and visual^15–17^ manifestations of COVID-19 since the start of the pandemic, the impact of the disease on the auditory and vestibular systems has received little attention so far. In addition, although three reviews (including two systematic reviews^7,9^ and one narrative review^18^ on a few papers, predominately case reports/series) over auditory and vestibular symptoms of SARS-CoV-2 were published, no meta-analysis paper is available yet. The present paper intends to systematically review current evidence regarding hearing loss, tinnitus, and dizziness caused by SARS-CoV-2, as well as determine the occurrence frequency of these symptoms through a meta-analysis.

## Methods

### Search Strategy

The present systematic review was conducted based on the guidelines of the Preferred Reporting Items for Systematic Reviews and Meta-Analysis (PRISMA).^19^ A comprehensive search was conducted on electronic healthcare databases including PubMed, ScienceDirect, and Wiley, as well as WHO updates in Oct 2020, updated Jan 2021, using combined keywords: ‘COVID-19,’ ‘SARS-CoV-2,’ ‘pandemic,’ ‘auditory dysfunction,’ ‘hearing loss,’ ‘tinnitus,’ ‘vestibular dysfunction,’ ‘dizziness,’ ‘vertigo,’ and ‘otologic symptoms.’

#### Inclusion/Exclusion Criteria

Cross-sectional, cohort, retrospective, or case-control studies in the English language reporting hearing loss, tinnitus, and/or dizziness on confirmed cases with COVID-19 were considered for the review. Reviews, books, case reports, case series, letters, editorials, and notes/commentaries were excluded.

The duplicates were eliminated using Endnote software (Thomson Reuters, Philadelphia, Pennsylvania, USA, version X7). The final papers were included through a three-stage process: title screening, abstract screening, and full-text screening. In each section, the papers that did not meet the inclusion criteria were excluded. In the cases of uncertainty, first the abstracts and then the full texts were screened. All three stages were independently conducted by two reviewers. If there was any disagreement, the authors discussed to reach a consensus. Overall, there was a complete agreement between the reviewers.

### Quality Assessment

The Crowe Critical Appraisal Tool (CCAT) was applied for quality measurement.^20^ The CCAT is one of the few instruments that has undergone both reliability and validity evaluations and is used to appraise different research study designs.^20,21^ The tool assesses the following eight aspects of each paper: preliminaries, introduction, design, sampling, data collection, ethical issues, results/findings, and discussion using a six-point scale (from 0 to 5 for each category) with a total potential score of 40.

### Data Analysis

The meta-analysis was carried out using Comprehensive Meta-Analysis Version 3.0 (Biostat, Inc. Englewood, NJ, USA) to investigate whether the event rate (the occurrence frequency of an event) of hearing loss (*n* = 4 studies),^11,22–24^ tinnitus (*n* = 6 studies),^11,22–26^ and dizziness (*n* = 9 studies)^11–13,24,25,27–30^ is statistically significant. To prevent confounding bias resulting from low sample sizes, the papers with a sample size fewer than 50 were not considered in the analysis.^31–33^ Heterogeneity was assessed using the Q-Cochrane test and *I*-squared (*I*2, I: inconsistency index) statistic. Whenever heterogeneity was confirmed (*I* ^2^ value> 0.75 and *P*-value< 0.1 through Q-Cochrane test), a random-effect meta-analysis with 95% confidence intervals (CIs) was performed; otherwise, a fixed-effect meta-analysis was applied.^34^ Forest plots were used to present the pooled estimates of ERs and 95% CIs.

## Results

### The Systematic Review Results

**Figure 1:**
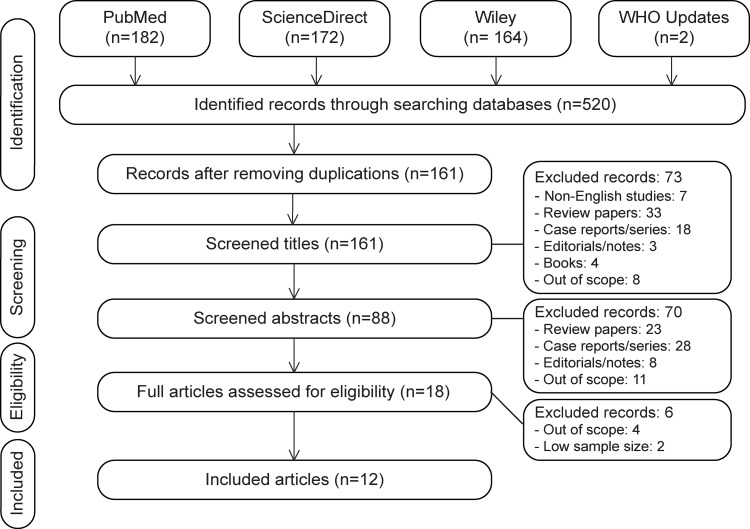
PRISMA flow diagram demonstrating the summary of literature search and screening process. PRISMA, preferred reporting items for systematic reviews and meta-analyses.

The database search yielded 520 papers (Figure 1). Duplicated papers (*n* = 359), non-English papers (*n* = 7), reviews (*n* = 33), case reports/case series (*n* = 18), editorials/notes/commentaries (*n* = 3), books (*n* = 4), and papers out of the study scope (*n* = 8) were removed. This initial screening resulted in a set of 161 papers, which were evaluated to extract the papers corresponding with the inclusion criteria. Seventy more papers were eliminated during the screening of papers’ abstracts (e.g., reviews = 23, case reports/case series = 28 (Table 1),^35–62^ editorials/notes/commentaries = 8, and out of the scope = 11). Among 18 studies extracted for the full-text review, 6 papers were removed (e.g., out of the scope = 4 and with low sample size=2) and 12 papers were selected for final statistical analysis (Table 2, Figure 1). The ERs of hearing loss, tinnitus, and dizziness were collected from four,^11,22–24^ six,^11,22–26^ and nine^11–13,24,25,27–30^ papers, respectively. The reference lists for the selected publications were also hand-searched for any additional related publications. No additional related article, however, was found. The bias resulted from only searching databases in the English language (language bias) is acknowledged.

**Table 1:** Case reports/series reporting hearing loss, tinnitus, and/or dizziness in confirmed patients with COVID-19

Study	Case(s)	Major symptom(s)	Study design	Findings
Chirakkal et al. (2021)^60^	1, 35yr, female	HL and tinnitus	Case report	Unilateral SNHL and tinnitus
Talebi Bezmin Abadi et al. (2021)^62^	1, 29yr, male	Dizziness	Case report	Dizziness and imbalance
Chern et al. (2021)^59^	1, 18yr, female	Sudden HL, tinnitus, vertigo	Case report	Sudden SNHL, aural fullness, vertigo, and intralabyrinthine hemorrhage
Gunay et al. (2021)^61^	1, 23yr, female	Sudden HL and ear pain	Case report	Bilateral mixed hearing loss and ear pain
Kanjwal et al. (2020)^43^	1, 36yr, female	Dizziness	Case report	Dizziness, presyncope, fatigue, and orthostatic palpitations
Woodhall et al. (2020)^57^	1, 39yr, female	Dizziness	Case report	Dizziness and visual loss
Avener et al. (2020)^36^	1, 88yr, female	Dizziness	Case report	Dizziness, syncope, slurred speech, and confusion
Lamounier et al. (2020)^48^	1, 67yr, female	Sudden HL and tinnitus	Case report	Sudden SNHL (severe in the right ear and mild in the left ear) and disabling tinnitus
Fadakar et al. (2020)^39^	1, 47yr, male	Vertigo	Case report	Progressive vertigo and ataxia
Koumpa et al. (2020)^47^	1, 45yr	Sudden HL and tinnitus	Case report	Unilateral sudden severe high-frequency SNHL and tinnitus
Lang et al. (2020)^49^	1, 30yr female	Sudden HL and tinnitus	Case report	Unilateral sudden severe to profound high-frequency SNHL and tinnitus
Abdel Rahman and Abdel Wahid (2020)^35^	1, 52yr male	Sudden HL and tinnitus	Case report	Unilateral sudden severe to profound SNHL and tinnitus
Degen et al. (2020)^38^	1, 60yr, male	HL and tinnitus	Case report	Bilateral profound hearing loss with loud tinnitus
Kilic et al. (2020)^45^	5 males	HL	Case report	COVID-19 was positive in one of the cases.
Sia (2020)^53^	1, 78yr, male	Dizziness	Case report	Dizziness and unsteadiness
Jacob et al. (2020)^42^	1, 61yr, female	HL	Case report	Hearing loss
Chen et al. (2020)^37^	1, 34yr, male	Dizziness	Cases report	Dizziness
Karimi-Galougahi et al. (2020)^44^	6, 22–40yr, 4female, 5male	HL, tinnitus, Vertigo	Case series	Unilateral moderate to severe SNHL (*n* = 6), tinnitus (*n* = 4), vertigo (*n* = 2)
Malayala and Raza (2020)^51^	1, 29yr, female	Vertigo	Case report	Symptoms of acute vestibular neuritis intractable vertigo, nausea, and vomiting
Liu et al. (2020)^50^	1, 37yr, male	Vertigo	Case report	Vertigo
Takahashi et al. (2020)^56^	1, 73yr, male	HL	Case report	Sudden hearing loss
Han et al. (2020)^41^	1, 47yr, male	Vertigo	Case report	Vertigo
Sun et al. (2020)^55^	1, 38yr, male	HL and tinnitus	Case report	Bilateral hearing loss and tinnitus
Norman et al. (2020)^52^	1, 83yr, female	Dizziness	Case report	Dizziness
Sriwijitalai and Wiwanitkit (2020)^54^	1 old female	HL	Case report	SNHL
Kong et al. (2020)^46^	1, 53yr, female	Dizziness	Case report	Sudden dizziness and symptoms of the nervous system involvement
Fidan (2020)^40^	1, 35yr, female	HL and tinnitus	Case report	Unilateral mild-to-moderate CHL due to acute otitis media with otalgia and tinnitus
Wu et al. (2020)^58^	1, 41, male	Dizziness	Case report	Dizziness

CHL, conductive hearing loss; HL, hearing loss; SNHL, sensory-neural hearing loss.

**Table 2:** Studies included in the meta-analysis regarding hearing loss, tinnitus, and/or dizziness in confirmed patients with COVID-19

Study	Country	Sample size	Age (years)	Aim	Study design	Findings
Elibol (2021)^22^	Turkey	155	18–72	Frequency of otologic symptoms (64 hospitalized)	Retrospective, using medical records	HL: 1 Tinnitus: 2
Viola et al. (2020)^24^	Italy	185	52.15 (19–81)	Occurrence frequency of tinnitus and dizziness	Cross-sectional, using an online questionnaire	Tinnitus: 43 Dizziness: 34 (Dizziness: 32 and Vertigo: 2)
Özçelik Korkmaz et al. (2020)^11^	Turkey	116	57.24 (19–83)	Otolaryngologic manifestations of hospitalized patients with mild to moderate symptoms	Prospective, using a case history	HL: 6 Tinnitus: 13 Dizziness: 43 (Dizziness: 37 and Vertigo: 6)
Freni et al. (2020)^23^	Italy	50	37.7 (18–65)	Otolaryngologic manifestations of outpatients	Cross-sectional, using online standard questionnaires (e.g., HHI, THI)	Hl: 9 Tinnitus: 5
Lechien et al. (2020)^26^	Europe	1420	39.17	Clinical characteristics of cases with mild to moderate symptoms	Cross-sectional, using a questionnaire	Tinnitus: 5
Mao et al. (2020)^12^	China	214	52.7	Neurologic manifestations of hospitalized patients with severe condition	Retrospective, using medical records	Dizziness: 36
Karadaş et al. (2020)^25^	Turkey	239	46.56 (19–88)	Neurologic manifestations of hospitalized patients	Prospective, using neurological examinations and medical records	HL: 3 Tinnitus: 5 Dizziness: 22 (Dizziness: 16 and Disequilibrium: 6)
Vacchiano et al. (2020)^29^	Italy	108	59 (18–83)	Early neurological manifestations of hospitalized patients	Cross-sectional, using a questionnaire	Dizziness: 11
Chen T et al. (2020)^28^	China	799	68 (62–77)	Clinical characteristics of hospitalized patients with severe or critical symptoms	Retrospective, using medical records	Dizziness: 21
Wang Z et al. (2020)^30^	China	69	42 (35–62)	Clinical characteristics of hospitalized patients	Retrospective, using medical records	Dizziness: 5
Chen Q et al. (2020)^27^	China	145	47.5	Clinical characteristics of hospitalized patients	Retrospective, using medical records	Dizziness: 29
Wang D et al. (2020)^13^	China	138	56 (42–68)	Clinical characteristics of hospitalized patients with mild to severe symptoms	Retrospective, using medical records	Dizziness: 13

HL: hearing loss; HHIE: hearing handicap inventory; THI: tinnitus handicap inventory.

#### The Strength of the Evidence

The included studies were assessed for methodological quality using CCAT^63,64^ (Table 3). According to CCAT recommendations, the scores were reported as both a total score and a percentage. The quality of the studies rated between 50 and 72.5% (mean: 62.3 %). Overall, only one study scored above 70%.^26^ Among the 12 studies included, the study design was single group prospective (*n* = 2),^11,25^ cross-sectional (*n* = 4),^23,24,26,29^ or retrospective (*n* = 6)^12,13,22,27,28,30^ (Table 2). None of the studies used objective standard tests to access hearing loss, tinnitus, or dizziness, and the data collection was performed subjectively through using case history forms, questionnaires, or medical records. Only one study applied standard questionnaires to collect information about hearing loss and tinnitus (i.e., using hearing handicap inventory (HHI) and tinnitus handicap inventory (THI)).^23^ The studies were deficient in other aspects, including sampling (e.g., sample size calculations and/or inclusion/exclusion criteria) and poor or not reporting ethical considerations (e.g., ethical approval).

**Table 3:** Quality assessment of the included papers using Crowe critical appraisal tool (CCAT)

Study	Preliminaries	Introduction	Design	Sampling	Data collection	Ethics	Results	Discussion	Total/40	Total (%)
Elibol (2021)^22^	3	2	3	3	2	4	3	3	23/40	57.5
Viola et al. (2020)^24^	3	2	2	2	2	3	3	3	20/40	50.0
Özçelik Korkmaz et al. (2020)^11^	3	3	3	3	3	2	4	4	25/40	62.5
Freni et al. (2020)^23^	4	4	3	3	3	2	4	4	27/40	67.5
Lechien et al. (2020)^26^	3	3	3	4	3	5	4	4	29/40	72.5
Mao et al. (2020)^12^	4	3	4	3	3	3	4	3	27/40	67.5
Karadaş et al. (2020)^25^	3	4	3	3	2	5	3	3	26/40	65.0
Vacchiano et al. (2020)^29^	3	2	2	2	2	5	2	2	20/40	50
Chen T et al. (2020)^28^	4	4	3	4	3	0	4	4	26/40	65
Wang Z et al. (2020)^30^	3	3	3	2	3	0	4	4	22/40	55
Chen Q et al. (2020)^27^	2	3	4	3	2	5	4	4	27/40	67.5
Wang D et al. (2020)^13^	4	4	3	3	3	2	4	4	27/40	67.5

### The Meta-Analysis Results

#### Hearing Loss

**Figure 2:**
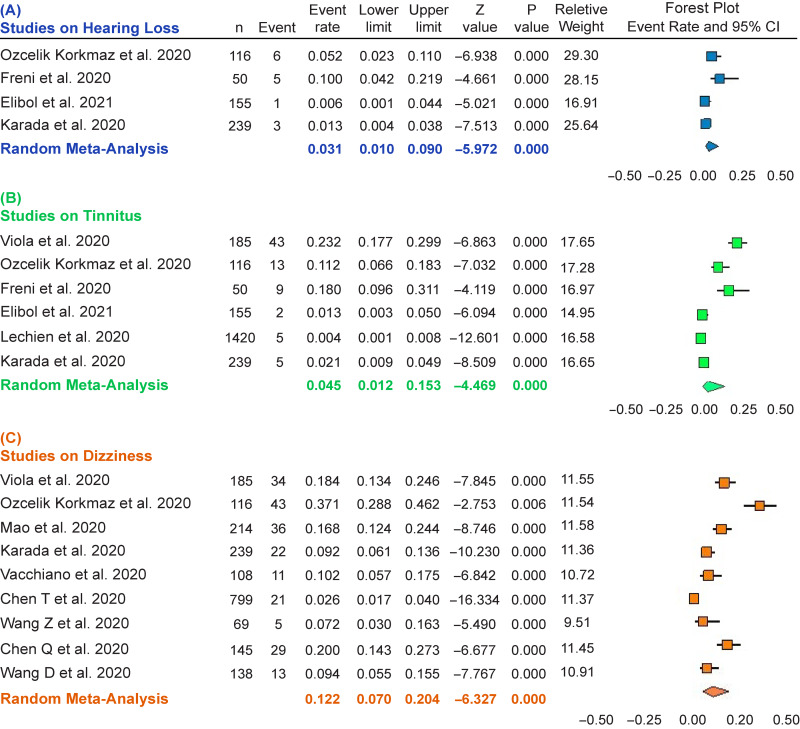
Forest plot for the event rate of hearing loss in four studies (A), tinnitus in six studies (B), and dizziness in nine studies (C). The square size indicates the statistical weight for each study. The horizontal line represents 95% CI, and the diamond summarizes the overall estimate of event rate and its corresponding 95% CI. CI, confidence interval.

Given the extent of heterogeneity (i.e., *I* ^2^ = 75.661, P = 0.006), a random meta-analysis was performed on four papers^11,22–24^ (Table 2) reporting hearing loss in cases with COVID-19. The total population was equal to 560, and the sample size varied between 50 and 239 (Figure 2A). The ER of hearing loss was equal to 0.031 with a CI between 0.01 and 0.09 (df = 3, *Z*: −5.972, *P* ≤ 0.001).

#### Tinnitus

A random meta-analysis (*I* ^2^ = 95.718, *P* ≤ 0.001) was conducted on six papers^11,22–26^ (Table 2) reporting tinnitus occurrence in cases with COVID-19. The total population was equivalent to 2165, and the sample size varied between 50 and 1420 (Figure 2B). The ER of tinnitus was obtained 0.045 with a CI between 0.012 and 0.153 (df = 5, *Z* = -4.469, *P* ≤ 0.001).

#### Dizziness

A random meta-analysis (*I* ^2^ = 93.846, *P* ≤ 0.001) was carried out on nine papers^11–13,24,25,27–30^ (Table 2) reporting dizziness occurrence in patients with COVID-19. The total population was equal to 2013, and the sample size varied between 69 and 239 (Figure 2C). The ER of dizziness was obtained 0.122 with a CI: 0.070–0.204 (df = 8, *Z* = -6.327, *P* ≤ 0.001).

In each forest plot in Figure 2, each horizontal line drawn onto a forest plot demonstrates a separate study being analyzed. The result of each study includes two components comprising: (1) a black box indicating a point estimate of ER and (2) a horizontal line representing the 95% CIs of the study result.^65^ For example, in Figure 2A, the point estimate of ER in the Özçelik Korkmaz et al. (2020) study is 0.052, and its CI is between 0.023 and 0.110. The weight (%) represents the impact of each study on the pooled result. For instance, in Figure 2A, the Özçelik Korkmaz et al. (2020) study and the Elibol (2021) study had the highest and lowest weights, respectively. A diamond below each forest plot exhibits the overall point estimate of ER and its CIs obtained by meta-analysis (e.g., ER = 0.031 and CIs: 0.010–0.090 in Figure 2A). The center of the diamond displays the pooled point estimate, and its horizontal tips show the 95% CIs.^65^

## Discussion

### Hearing Loss in Patients with COVID-19

The present meta-analysis on four studies^11,22–24^ shows the occurrence rate of 3.10% (CIs: 0.010–0.090) or 3.1% for hearing loss on confirmed cases with COVID-19. This number, however, should be interpreted with precaution because of the low level of evidence (e.g., studies with no control group) and high heterogeneity among the papers.^66^ Given the subjective nature of data collection, the studies also provide no evidence considering the severity (i.e., slight to profound) and the type of the hearing loss (i.e., sensory-neural hearing loss (SNHL), conductive hearing loss (CHL), or mixed hearing loss), as well as the potential pathology involved. In descriptive or self-report studies, it is also likely that slight to mild changes in hearing ability are being ignored, especially in patients with a severe condition. In this regard, current publications over the occurrence frequency of olfactory dysfunction in patients with COVID-19 demonstrate a remarkable difference between the results of using standard objective tests compared with subjective data collection (e.g., 86.6% vs. 36.64%).^14^ Past research also points to the discrepancy between findings of objective hearing assessments and subjective self-reports.^67,68^

In terms of the potential type of hearing loss, case series^44,45^ and case reports^35,38,47,49,51^ using objective hearing assessments have predominantly reported SNHL (Table 1), which may result from the direct impact of SARS-CoV-2 on the organ of Corti, stria vascularis, and/or spiral ganglion.^69,70^ For instance, in three case reports^35,47,49^ and one case series,^44^ unilateral sudden moderate to profound high-frequency SNHL and tinnitus, with no or partial improvement following intratympanic steroid administration, were reported.^35,47,49^ Sudden SNHL is characterized as SNHL of 30 dB or greater in at least three consecutive frequencies over 72 h.^45^ This type of hearing loss is a known complication of several viral infections, which can damage the inner ear structures or accelerate inflammatory processes leading to sudden SNHL.^49^ Only in one case report, unilateral CHL with otalgia and tinnitus was observed.^40^

### Tinnitus in Patients with COVID-19

Tinnitus is defined as the sensation of sound without any external acoustic source (phantom perception of sound). It shows the prevalence of 10%–15% in the adult population and can be identified by self-report or using case history forms/self-assessment questionnaires.^71,72^ Cochlear abnormalities produced by known risk factors (e.g., long-term noise exposure, ototoxic drugs, aging, and genetic predispositions) and concomitant neural alterations are considered as the initial source of tinnitus.^73^ Our meta-analysis on six papers demonstrates the occurrence rate of 4.50% (CIs: 0.012–0.153) for tinnitus in patients with COVID-19. This finding may result from the impact of SARS-CoV-2 on the auditory system and/or point to the mental or emotional burden of the pandemic.^74^ The reviewed studies, however, presented no further information considering the tinnitus sound (i.e., how it looks like), loudness (e.g., soft to loud), place (e.g., unilateral or bilateral), severity (e.g., slight to catastrophic handicap), and duration (e.g., intermittent or constant), which could be helpful to further interpret the results.

In three questionnaire-based studies on individuals with tinnitus (without COVID-19) to examine the pandemic mental burden on tinnitus perception, an increase in tinnitus-related handicap and distress was shown in those who perceived the situation stressful and bothersome (Table 4).^74–76^ In one of these studies using data collected by an online survey among 3,103 individuals with tinnitus from several countries, emotional consequences of the pandemic were associated with tinnitus exacerbation in 42% of participants, especially for those who were self-isolated, alone, and/or with sleep difficulties and reduced physical activity.^74^ This finding is in line with studies that demonstrate the contribution of environmental factors in modulating tinnitus.^73,77^

**Table 4:** The pandemic impact on tinnitus severity and distress in individuals without COVID-19

Study	Country	Sample size	Age (years)	Study design	Findings
Beukes et al. (2020)^74^	48 countries	3103	58 (18–100)	Cross-sectional using questionnaires	Seven new cases with tinnitus, 42% tinnitus exacerbation, and 32% more bothersome tinnitus, especially in self-isolated, alone individuals with poor sleep.
Schlee et al. (2020)^75^	Germany	122	54	Pre/post-study using questionnaires (THI, TQ, MDI, BFI2, and SOISES)	Increased tinnitus distress in people who perceived the situation stressful with increased grief, frustration, stress, and nervousness.
Anzivino et al. (2021)^76^	Italy	16	Adults	Pr/post-study using a questionnaire (THI)	One level increase in the grade of tinnitus-related handicap in 12 out of 16 cases.

BFI2, big five index-2; MDI, major depression inventory; SOISES, social isolation electronic survey; THI, tinnitus handicap inventory; TQ, tinnitus questionnaire.

### Dizziness in Patients with COVID-19

Dizziness is a general term in medical diagnosis, which is traditionally classified into four categories given the patient’s history, including vertigo, disequilibrium, presyncope, and light-headedness.^78,79^ In vertigo (45%–54%), the patient perceives a false sensation of whirling or rotation originating from the vestibular system. Vertigo is mainly caused by benign paroxysmal positional vertigo, Ménière’s disease, vestibular neuritis, and labyrinthitis.^80^ Disequilibrium, feeling off-balance or wobbly, is more reported in patients with Parkinson’s disease and diabetic neuropathy (up to 16%). Many medications can cause presyncope, feeling of losing consciousness, or blacking out (up to 14%). Light-headedness, vague symptoms such as feeling disconnected from the environment, is commonly associated with psychogenic or psychiatric origins such as anxiety, depression, and somatoform disorders (10%–20%).^78,81^ Light-headedness and presyncope often overlap subjectively and are difficult to differentiate unless looking at duration.^82^

**Figure 3:**
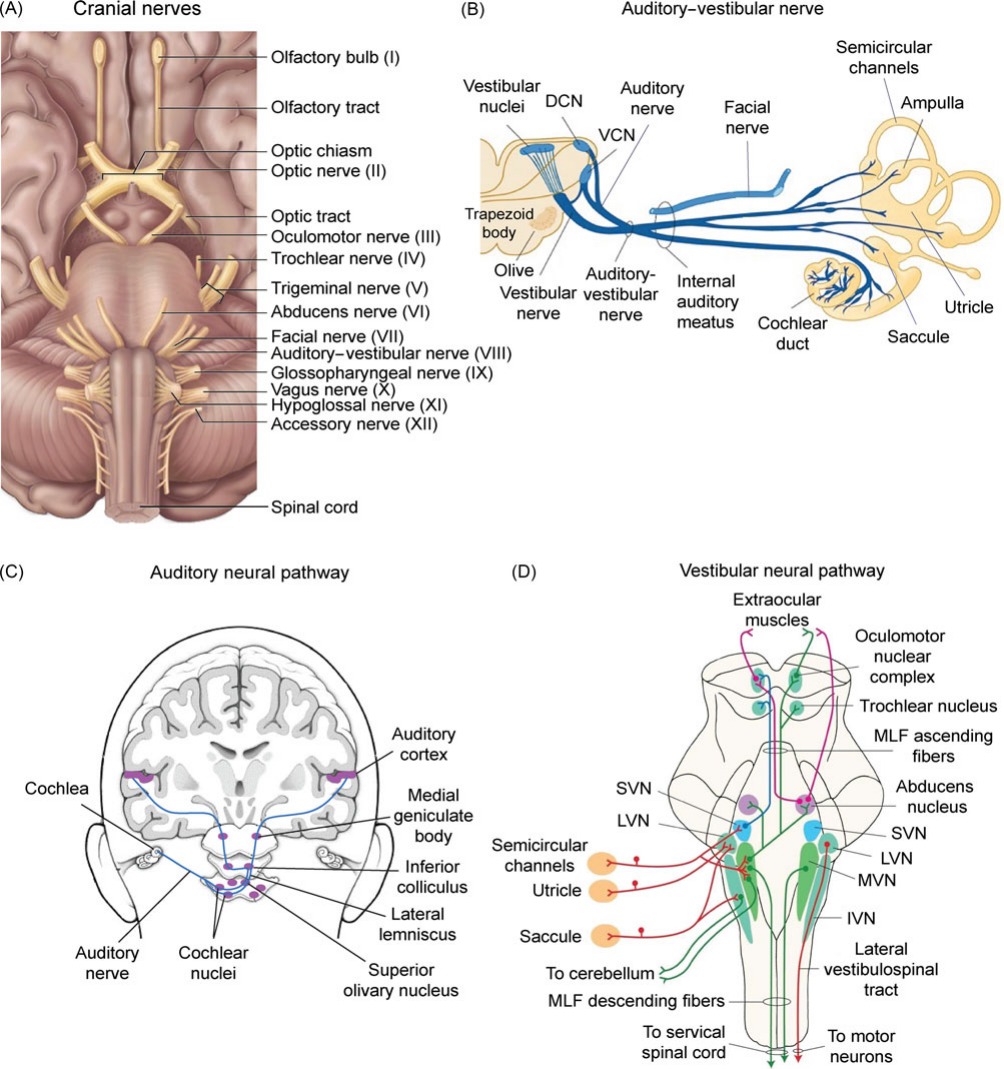
*Auditory and vestibular neural pathways in humans.* (A) Cranial nerves (I–XII) at the base of the brain. (B) Innervation of the cochlea and vestibule (i.e., semicircular channels, utricle, and saccule) by auditory and vestibular branches of the auditory–vestibular nerve (cranial nerve VIII). (C) The major ascending auditory neural pathways. (D) Vestibular neural pathways. The afferent fibers from the vestibular labyrinth project to each of the vestibular nuclei located in the rostral medulla and the caudal pons. Neural fibers from semicircular canals project to the SVN and rostral portion of the MVN. Neural fibers from the utricle and saccule terminate in LVN. Some saccular neural fibers project to the IVN. DCN, dorsal cochlear nucleus; IVN, inferior vestibular nucleus; LVN, lateral vestibular nucleus; MLF, medial longitudinal fasciculus; MVN, medial vestibular nucleus; SVN, superior vestibular nerve; VCN, ventral cochlear nucleus.

The present meta-analysis on nine papers demonstrates the occurrence rate of 12.20% (CIs: 0.070–0.204) for dizziness in cases with COVID-19. It has been shown that the inner ear structures are particularly susceptible to ischemia and vascular damage, which can lead to both hearing and balance dysfunction.^24^ Vasculitis also is characterized as one of the clinical manifestations of COVID-19.^83^ Evidence of dizziness/vertigo in patients with COVID-19 also has been raised in case reports.^37,41,50^ For instance, in a recent case study, a young female with COVID-19 was diagnosed with acute vestibular neuritis.^51^ Vestibular neuritis or acute peripheral vestibulopathy is a viral or post-viral inflammatory disease, which involves the vestibular part of the eighth cranial nerve^84^ (Figure 3A and B). The disease is clinically diagnosed with vertigo and develops acutely over minutes to hours. Whereas vestibular neuritis is generally considered to be a monophasic condition, multiple cranial nerve involvement also is likely in viral inflammation.^84^ The patient presented symptoms of intractable vertigo accompanied by nausea and vomiting, possibly due to irritation/deafferentation of the emetic tracts associated with the vestibular nerve/nuclei.^85^ Overall, likewise hearing loss and tinnitus, the occurrence frequency of dizziness in this study should be interpreted with precaution given the low level of evidence, heterogeneity among studies, and the lack of using standard objective tests for dizziness assessments.

In a recent systematic review and meta-analysis on audiovisual symptoms of COVID-19, the pooled estimate of the prevalence of hearing loss, tinnitus, and rotatory vertigo was reported as 7.6% (CIs: 2.5–15.1), 14.8% (CIs: 6.3–26.1), and 7.2% (CIs: 0.01–26.4), respectively.^86^ The estimated prevalence values obtained in our meta-analysis fall within the lower end of the CIs in this study, which may result from the difference between the two studies in inclusion/exclusion criteria. For instance, Almufarrij and Munro (2021) considered no restriction in terms of the diagnostic tool used to detect SARS-CoV-2 and included studies on probable (i.e., medically confirmed) and suspected (i.e., medically unconfirmed) cases with COVID-19 too. But in the present meta-analysis, only studies on patients with confirmed PCR test results were included. The papers with a sample size less than 50 were also excluded in our study to prevent confounding bias associated with low sample sizes.

### Potential Pathophysiology and Mechanisms

#### Brainstem Damage

The auditory and vestibular systems are two sensory systems that are mostly present in the brainstem (Figure 3C and D). Auditory inputs are transferred from the auditory branch of the eighth cranial nerve to the cochlear nuclei, lateral lemniscus, inferior colliculus, and medial geniculate body before projection to the auditory cortex.^87^ The vestibular branch of the eighth cranial nerve also conveys vestibular inputs to the vestibular nuclei (Figure 3D) that in turn project to the thalamus. Multiple thalamic nuclei contribute to vestibular processing, which contain multisensory neurons and process vestibular, proprioceptive, and visual signals and project to the cortex.^88^ The brainstem also controls the sleep–wake cycle and vital functions through the ascending reticular activating system and the autonomic nuclei, respectively. Thus, brainstem dysfunction resulting from neuroinflammatory mechanisms triggered by SARS-CoV-2 can produce sensory (including auditory and vestibular) and motor deficits, cranial nerve palsies, impairment of consciousness, dysautonomia, and respiratory failure.^89^

#### Inflammatory Mechanisms

Inflammation is a natural defense mechanism against pathogens and involves many pathogenic diseases such as microbial and viral infections, as well as autoimmune and chronic diseases.^90^ Oxidative stress also refers to the excessive production of reactive oxygen species (ROS) in cells and tissues, which can impair cellular molecules such as DNA, proteins, and lipids. ROS is implicated in the regulation of processes involved in cell homeostasis and functions and is normally produced in limited quantities in the body.^91^ Excessive ROS and some natural or artificial chemicals can stimulate inflammatory processes and lead to the synthesis and secretion of proinflammatory cytokines (e.g., Interleukin 6 (IL-6), IL-1β) and tumor necrosis factor–alpha (TNF-α). Inflammation and oxidative stress are closely associated with pathophysiological processes and are tightly linked to one another. Thus, the activation of both processes is simultaneously found in many pathological conditions, including infection with SARS-CoV-2.^92^ Past studies also show the contribution of ROS and proinflammatory cytokines in initiating acute and chronic inflammation in SNHL and tinnitus,^93,94^ in which they also may play a role in damaging the inner ear in patients with COVID-19.^95^ Given that SARS-CoV-2 is linked to an intense systemic immune reaction, called a ‘cytokine storm,’ the overreaction of microglia also is a possible trigger for postinfectious neuroinflammation, which also may play a role in damage to the auditory glial cells.^96^

#### Hematogenous Track

Findings of studies demonstrate that SARS-CoV-2 can attach to the hemoglobin and penetrates the erythrocyte. Therefore, it can be transported with erythrocytes or vascular endothelium to all the tissues with ACE2 in their structure, including the brain and medulla oblongata that have plenty of ACE2, as well as the auditory system.^97^ Although the expression of the ACE2 gene was shown in the mouse cochlea,^98^ the presence of SARS-CoV-2 in the human inner ear has not been reported yet. Human evidence demonstrates that SARS-CoV-2 can spread throughout the body via the circulation system because of the abundant expression of ACE2 in arterial and venous endothelial cells and arterial smooth muscle cells in many organs.^99^ The virus may also damage the blood–labyrinth barrier and invade the inner ear structure by infected and activated monocytes due to attack of the vascular system.^100^ The process of deoxygenation of erythrocytes by the virus also can lead to hypoxia and further damage to the inner ear.^101^ Hypoxia even may occur in cases with no COVID-19 symptoms. For instance, some patients may present significantly reduced pulse oximetry reading, which is called ‘silent’ or ‘apathetic hypoxia,’ despite having no or minimal symptoms.^102^

### Ototoxicity by Antiviral Treatments

Remdesivir, ribavirin, and synthetic quinine products (chloroquine (CQ) and hydroxychloroquine (HCQ) are traditionally used for the treatment of malaria, autoimmune diseases, and systemic erythematosus lupus given their antiviral and anti-inflammatory properties.^8^ According to a new update on a large-scale randomized-control study at 405 hospitals in 30 countries by WHO Solidarity Trial Consortium, these medications have little or no effect on hospitalized patients with COVID-19, in terms of overall mortality, initiation of ventilation, and duration of hospital stay.^103^ CQ and HCQ also show several side effects, such as ototoxicity, retinopathy, neuromyopathy, and cardiomyopathy. For instance, they can cause temporary or permanent auditory toxicity and lead to SNHL and tinnitus in both acute and chronic consumption, which has been reported is irreversible following CQ.^104^ This ototoxicity potentially results from damage to the inner ear and neural organs such as outer hair cells, spiral cell ganglions, neural fibers, the atrophy of stria vascularis, as well as changes in the central auditory system.^8^ Additionally, CQ can increase the glutamate concentration in the extracellular environment and causes ROS overproduction, which is another known mediator of neural toxicity in the glial cells of the inner ear.^105^

## Conclusions

Twelve single-group prospective, cross-sectional, or retrospective studies on confirmed patients with COVID-19 were reviewed in this study. Except for four studies on otolaryngologic manifestations of SARS-CoV-2, other studies were more general, over neurological or clinical characteristics of the disease. Our meta-analysis demonstrates that the occurrence rate of hearing loss (0.031), tinnitus (0.045), and dizziness (0.122) is statistically significant in patients with COVID-19. But, given the low level of evidence (i.e., studies with no control group), weakness in data collection (i.e., using self-reports and/or medical records), and high heterogeneity among studies reviewed, these results should be interpreted with caution. Weakness in data collection also may contribute to the magnitude of ERs, especially in cases with mild symptoms or those in a critical condition. Thus, well-designed studies and follow-up assessments using standard objective tests are necessary, which can provide more precise information given the occurrence frequency, type, and severity of otologic symptoms (hearing loss, tinnitus, and dizziness); the rate of improvement after recovery; and the link between disease severity and the auditory–vestibular involvement.
